# Impact of *Avibacterium paragallinarum* infection on the upper respiratory tract microbiome of chickens

**DOI:** 10.3389/fvets.2026.1911133

**Published:** 2026-09-21

**Authors:** Madhusudan Timilsina, Dhiraj Chundru, Alyssa Harris, Mostafa Ghanem

**Affiliations:** Department of Veterinary Medicine, University of Maryland, College Park, MD, United States

**Keywords:** 16s sequencing, chicken, infectious coryza, microbial diversity, upper respiratory microbiome

## Abstract

*Avibacterium paragallinarum*, the causative agent of infectious coryza, causes infection in the upper respiratory tract (URT) of chickens, leading to substantial economic losses. While respiratory pathogens are known to disrupt host-associated microbiomes, *A. paragallinarum’s* impact on the chicken URT microbiome remains poorly understood. This study characterized microbiome changes in chicken URT following *A. paragallinarum* infection and examined whether different field strains induced distinct alterations. Four field strains were used to challenge separate groups of four-week-old specific-pathogen-free chickens, with one uninfected control group. Choanal swabs were collected at multiple time points post-challenge, pooled by group, and subjected to 16S rRNA V3-V4 amplicon sequencing. Microbial composition and diversity were characterized and compared among groups. Because of the limited biological replicates, statistical analyses and associated *p*-values were interpreted as exploratory. The URT microbiome was initially dominated by Firmicutes, followed by Proteobacteria, with the latter showing an increasing trend during infection. Infected group exhibited lower microbial richness compared to the noninfected based on the Chao1 index (*p* = 0.03175) and observed ASVs (*p* = 0.03372), with richness reaching the lowest level at 4 days post-inoculation (dpi) and returning to baseline by 9 dpi. Microbial community composition differed between infected and noninfected groups based on unweighted UniFrac distances (*p* = 0.027, PERMDISP ns). No differences in microbial diversity measures were observed among the individual strain groups. The findings suggest that *A. paragallinarum* infection is associated with transient yet notable alterations in microbial richness and community composition in URT, even in absence of overt clinical signs.

## 1 Introduction

*Avibacterium paragallinarum* is a Gram-negative, non-motile, rod-shaped, and bipolar staining bacteria of *Pasteurellaceae* family. It causes an acute and occasionally chronic infection of the upper respiratory tract (URT) of chickens, known as Infectious Coryza (IC). In general, chickens affected by IC show facial swelling, nasal discharge, lacrimation and reduced feed and water consumption resulting in reduced growth rate in growing chickens and decreased egg production in laying chickens (1). It results in economic losses primarily due to growth retardation and increased condemnation in broilers, decreased egg production in laying chickens, and increased cost related to biosecurity and disease management (2). Susceptible birds contract the disease via inhalation of infected aerosols or ingestion of contaminated feed and water. Antibiotics used for controlling IC reduces the clinical signs of the disease but there is continued colonization of the URT of the chickens by the bacteria after the discontinuation of the antibiotics (3, 4).

The chicken URT is a critical interface between the host and the external environment, and the resident microbial communities interact closely with the mucosal immune system to influence respiratory health, disease susceptibility, and pathogen colonization (5). The avian URT is known to have a rich and diverse microbial community (6), and its stability is essential for maintaining poultry health (7). Resident microbiota maintain host health through resistance to pathogen colonization, improvement of mucosal immunity, and act as a gatekeeper protecting the lower respiratory tract (5, 8).

Clinical disease is not always evident in many cases of URT infection in poultry including *Mycoplasmosis*, *Ornithobacteriosis* and *Infectious Coryza*, as it involves complex interaction between host, pathogen, environment, and presence of other co-infecting pathogens (6). However, the presence of other pathogens in the respiratory tract might intensify the clinical signs, resulting in a disease complex with higher severity, morbidity or mortality in the flock. Co-infection of *A. paragallinarum* with other respiratory pathogens such as *Mycoplasma gallisepticum* (9, 10), *Ornithobacterium rhinotracheale* (11), *Gallibacterium anatis* (12), Avian Influenza Virus (13), Fowl Adenovirus (14), Infectious Bronchitis Virus (15) is known to result in more severe disease in chickens. The composition and dynamics of microbiota in URT of chickens is, therefore, very important in the poultry health and productivity.

Several factors including the host and external environment are known to modulate the chicken URT microbiome, including age (7), housing conditions, heat stress, ammonia exposure (5), and litter management (16). Bacterial and viral infections also perturb the microbial ecosystem in the URT, as observed in infection by avian influenza virus (17, 18) and *Mycoplasma gallisepticum* (19). However, the impact of *A. paragallinarum* infection on the URT microbiome has not been systematically examined. Given the pathogen’s tropism for the URT epithelium and its ability to induce mucosal damage (20, 21), infection is likely to alter microbial community structure, with potential consequences for disease progression, recovery, and susceptibility to secondary pathogens. In addition, *A. paragallinarum* exhibits considerable genotypic (22) and phenotypic diversity, evident by the presence of NAD-independent strains (23), and recently the prevalence of atypical nonpathogenic strains in United States (24), which raises the possibility that different strains might affect the host health and microbiome differently.

In microbiome studies, microbial diversity is commonly evaluated using two complementary measures: alpha and beta diversity. Alpha diversity describes the diversity within a single sample or group, including microbial richness (number of taxa) and evenness (uniformity of taxa distribution). Beta diversity, in contrast, measures the compositional differences between two or more groups. In this study, we investigated the impact of *A. paragallinarum* infection on the URT microbiome of chickens using a controlled challenge model in four-week-old specific-pathogen-free birds. By comparing microbiome dynamics across multiple time points post-infection and among the experimental groups challenged with four field strains including two atypical strains, we aimed to explore the infection-associated microbial shifts and assess the contribution of strain-level variation. The findings and insights provide an initial characterization of microbiome changes associated with *A. paragallinarum* infection and may provide a basis for future studies investigating host-microbiome and pathogen interactions during infectious coryza and help toward the disease mitigation.

## 2 Materials and methods

### 2.1 Animal experiment and study design

The animal study consisted of five experimental groups of four-week-old specific pathogen free (SPF) White Leghorn chickens (*n* = 5 per group): one uninfected control group and four challenge groups, each inoculated with a distinct field strain of *A. paragallinarum*. The four challenge strains were previously isolated from commercial poultry flocks in the United States and represented different phenotypes and sequence types (STs) (22). AP10 was isolated from a clinical case in a commercial broiler flock from Delaware, AP36 and AP37 from *A. paragallinarum*-positive commercial layer flocks in Ohio with no clinical signs, and AP51 from a clinical case in commercial broiler flock from Ohio.

Chickens in the challenge groups were intranasally inoculated with 0.2 mL of bacterial suspension (10^7^ CFU/mL), while the control group received phosphate-buffered saline (PBS). Choanal swabs were collected from birds in each experimental group at days 0, 2, 4, 7, and 9 post-challenge and pooled by group at each timepoint longitudinally resulting in 25 samples (five groups × five timepoints). Clinical signs of the chickens were recorded and scored based on the previously described protocol; 0 for no signs, 1 for those with mild facial edema and nasal discharge, 2 for those with moderate facial edema and nasal discharge, and 3 for those with abundant nasal discharge, severe facial edema, and lacrimation (25). To confirm successful colonization, PCR targeting the HPG2 gene was performed on randomly selected birds from each challenge group (26). For microbiome analyses, samples collected after challenge from all bacterial challenge groups were additionally assigned as an “Infected” group. Samples from the PBS control group and samples collected at day 0 from the bacterial challenge groups, prior to inoculation, were combined into a “Noninfected” group.

### 2.2 DNA extraction and 16S rRNA gene amplification

Choanal swab samples were suspended in PBS, vortexed to release organic material, and centrifuged to pellet microbial biomass. DNA was extracted from the pellets using Qiagen Microbiome Kit (Qiagen, MD, United States) following the manufacturer’s protocol. DNA quantity and quality were assessed using spectrophotometric and fluorometric methods. The V3-V4 hypervariable region of the bacterial 16S rRNA gene was amplified using universal primers 341F (5′-CCTACGGGNGGCWGCAG-3′) and 785R (5′-GACTACHVG GGTATCTAATCC-3′). PCR reaction mixture was prepared in a total volume of 25 μL, constituting of GoTaq G2 Hot Start Master Mix (12.5 μL) (Promega, United States), DNase-free water (5 μL), forward primer (1.25 μL, 10 μM), reverse primer (1.25 μL, 10 μM), and DNA template (5 μL). PCR was carried out based on the following protocol: initial denaturation at 95 °C for 2 min, and 25 cycles of denaturation at 95 °C for 15 s, annealing at 60 °C for 30 s and extension at 72 °C for 45 s; and final extension at 72 °C for 10 min. Amplicon size and integrity were verified by agarose gel electrophoresis, followed by magnetic bead-based purification.

### 2.3 Library preparation and sequencing

A second PCR was performed to attach Illumina sequencing adapters and unique dual index sequences (IDT for Illumina) to the amplicons, enabling multiplexed sequencing. PCR was performed in 50 μL reaction volumes containing GoTaq G2 Hot Start polymerase (Promega, WI, United States), IDT for Illumina DNA/RNA UD Indexes (Illumina, San Diego CA), DNase-free water, and template DNA, following the Illumina-recommended cycling conditions: an initial denaturation at 95 °C for 3 min, 10 cycles of denaturation at 95 °C for 30 s, annealing at 57 °C for 30 s, and extension at 72 °C for 30 s, with a final extension at 72 °C for 5 min. The index-PCR products were cleaned up using CleanNGS magnetic beads (Bulldog Bio, NH, United States), and the fragment size of the prepared library was analyzed using Agilent 4,200 TapeStation (Agilent Technologies, CA, United States). Final concentration of the libraries was measured using Qubit fluorometer (Life Technologies, Paisley, United Kingdom), equimolarly pooled together, diluted to 4 nM, and spiked with 15% of PhiX. Paired-end sequencing of the samples was carried out on MiSeq platform using MiSeq v3 reagent cartridge (Illumina, San Diego CA).

### 2.4 Preprocessing and bioinformatic analysis of the sequence reads

Paired-end 16S V3-V4 sequence reads were processed in R-studio (v 2023.06.2 + 561) using Divisive Amplicon Denoising Algorithm 2 (DADA-2) package (*v1.29*)^1^ (27). Sequence quality profiles were inspected, followed by filtration and trimming of forward and reverse reads. Using DADA2, paired sequence reads were denoised and later merged together, followed by the chimeric sequence removal to generate an amplicon sequence variant (ASV) table. Taxonomic classification of the ASVs was performed using the *assignTaxonomy* function in the DADA2 package against the SILVA database (v 138.1) (28). Representative sequences of the ASVs were aligned using the *AlignSeqs* function of the DECIPHER package (v2.30.0). The aligned sequences were used to calculate a distance matrix and construct an initial neighbor-joining tree. The tree was subsequently optimized under a generalized time-reversible model with gamma-distributed rate variation and invariant sites (GTR + G + I) using the phangorn package (v2.12.1).

The ASV table, taxonomic assignments, phylogenetic tree, and the associated sample metadata were combined into a phyloseq object using the phyloseq package (v1.45.0) (29). Rarefaction curves were generated using the *rarecurve* function in the vegan package to assess ASV numbers across different sequencing depths. The dataset was subsequently rarefied to 90% of the minimum sample read count using the *rarefy_even_depth* function in the phyloseq package. Compositional difference between the different treatment groups was then evaluated using weighted and unweighted UniFrac-distances and visualized in Principal Coordinates analysis (PCoA) plots. Differences in alpha diversity among groups were evaluated using the Mann–Whitney U test and Kruskal-Wallis test, followed by Dunn’s *post hoc* test with Bonferroni correction for multiple comparisons. Differences in microbial community composition among treatment groups were assessed using permutational multivariate analysis of variance (PERMANOVA) implemented via *adonis2* function in the *vegan* package (30), and homogeneity of multivariate dispersions was evaluated using the PERMDISP. Linear Discriminant Analysis was conducted to identify differentially abundant taxa in the different treatment groups using *run_lefse* function in the microbiomeMarker package (v1.9.0) (31) with Kruskal-Wallis *p* < 0.05 and an LDA score ≥2 as significance thresholds. Because samples were pooled by treatment group at each timepoint, resulting in limited independent biological replicates, the interpretation of the statistical analyses and corresponding *p*-values should be considered exploratory.

## 3 Results

### 3.1 Experimental infection, sample collection, and sequence analysis

The animal experiment was conducted in an ABSL-2 + facility at the Department of Veterinary Medicine, University of Maryland, under Institutional Animal Care and Use Committee (IACUC) protocol R-FEB-22-07. None of the four field strains of *A. paragallinarum* induced clinical signs of infectious coryza in SPF chickens. Despite PCR confirmation of infection, all birds received a clinical sign score of zero. Grouped choanal swab samples were collected at 0, 2, 4, 7, and 9 dpi and analyzed using a 16S rRNA gene sequencing workflow to characterize changes in the respiratory microbiome following infection. Raw sequencing reads were processed using the *DADA2* pipeline. Primer sequences were removed (17 bp forward, 21 bp reverse), and reads were truncated at 285 bp and 195 bp position from the 5′ end, respectively. Sequences containing ambiguous bases, PhiX contamination, or more than two expected errors were discarded. Quality filtering and denoising reduced the median sequencing depth from 561,062 to 383,525 and subsequently to 382,987 reads per sample. Chimera removal further reduced median read counts to 354,674. Samples were rarefied to an even depth of 176,364 reads, corresponding to 90% of the sequencing depth of the sample with the lowest read count. Following rarefaction, and downstream processing using functions in the DADA2 package, a total of 1,760 amplicon sequence variants (ASVs) were identified across the 25 samples (Supplementary Figure S1).

### 3.2 *A. paragallinarum* infection associated with reduced microbial richness and altered community composition

*A. paragallinarum* infection was associated with reduced microbial richness in the upper respiratory tract. Both Chao1 index and Observed ASVs were lower in the infected groups compared with the noninfected groups (*p* = 0.03174 and *p* = 0.03372 respectively), whereas the Shannon index did not differ between the two groups (Figures 1A–C). PERMANOVA indicated differences in community composition based on the weighted (*p* = 0.013) and unweighted UniFrac distances (*p* = 0.027). However, the significant PERMDISP result for weighted UniFrac (*p* = 0.011) indicated heterogeneity in multivariate dispersion, suggesting that differences in within-group variability may have contributed to the significant PERMANOVA result and the result should be interpreted cautiously (Figures 1D,E). The *p*-values reported above are exploratory rather than confirmatory due to limited biological replicates.

**Figure 1:**
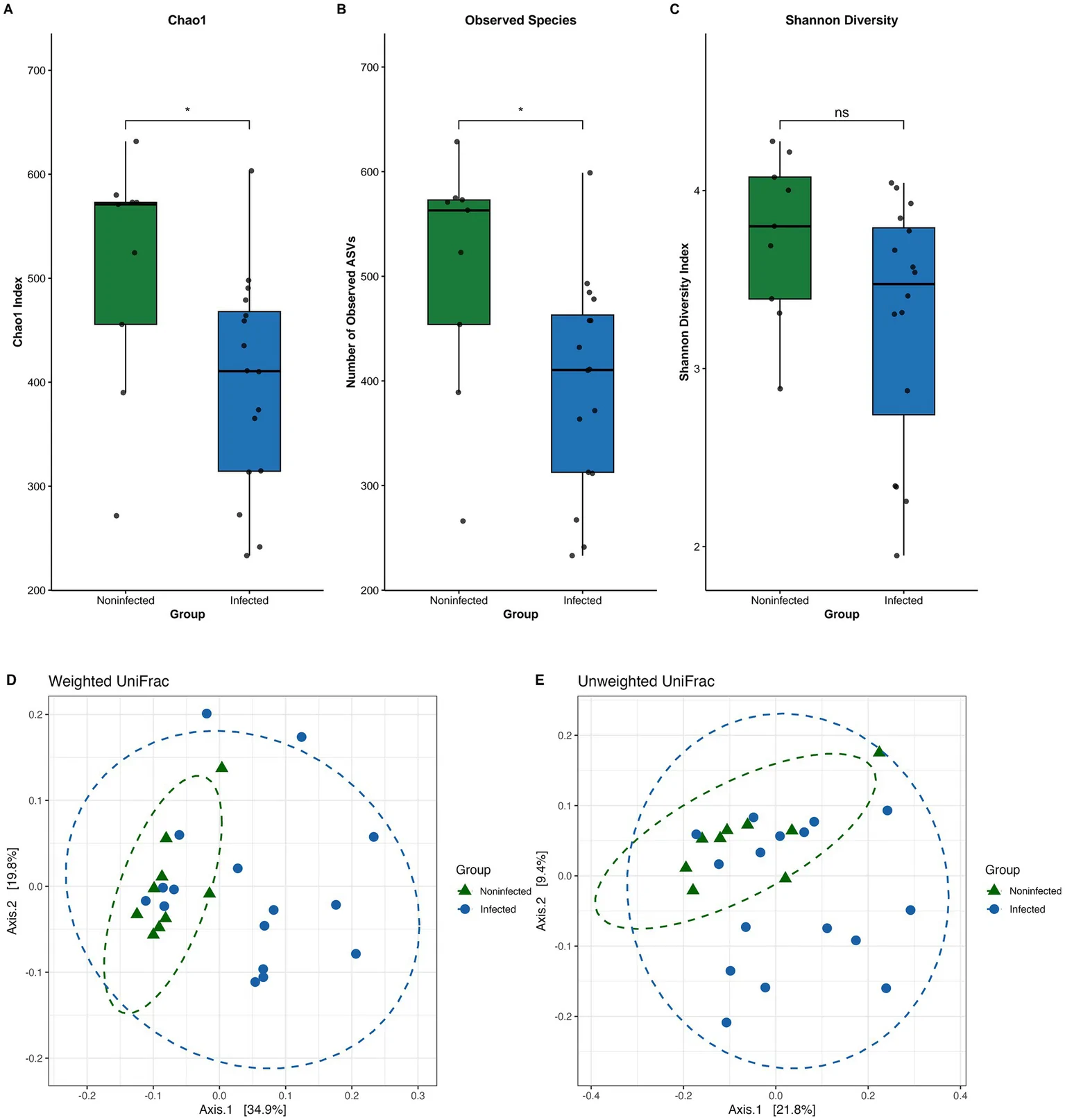
Alpha and beta diversity differences between noninfected and infected chickens. **(A–C)** Box plots comparing alpha diversity metrics between noninfected (green) and infected (blue) groups using the Chao1 index **(A)**, observed ASVs **(B)**, and Shannon diversity index **(C)**. Statistical significance was assessed using the Mann–Whitney *U* test (Chao1: *p* = 0.03174; Observed ASVs: *p* = 0.03372; Shannon: *p* = 0.08424). **(D,E)** Principal coordinates analysis (PCoA) plots comparing beta diversity between noninfected (green triangles) and infected (blue circles) groups based on weighted UniFrac **(D)** and unweighted UniFrac **(E)** distances. Group compositional differences were assessed using PERMANOVA (weighted UniFrac: *p* = 0.013; unweighted UniFrac: *p* = 0.027). Homogeneity of multivariate dispersion was evaluated using PERMDISP, which was significant for weighted UniFrac (*F* = 8.51, *p* = 0.011) but not for unweighted UniFrac (*F* = 2.91, *p* = 0.086). Given the limited number of independent replicates, the analysis and the corresponding *p*-values should be interpreted as exploratory.

### 3.3 Strain-associated differences on microbial community composition

Alpha diversity metrics, including Observed ASVs, Chao1, and Shannon diversity, did not differ among groups challenged with different *A. paragallinarum* strains (*p* > 0.05, each) (Supplementary Figures S2A–C). Furthermore, analysis of community composition across the five treatment groups using weighted and unweighted UniFrac distances indicated an overall effect of treatment (PERMANOVA, *p* < 0.05). However, pairwise comparisons after Bonferroni correction did not show differences in the microbial composition among the individual strain groups in the challenge experiment (*p* > 0.05, each) (Supplementary Figures S2D,E). Given the limited biological replicates, the *p*-values reported are hypothesis generating, rather than confirmatory.

### 3.4 Transient disruption and recovery of microbial richness following *A. paragallinarum* infection

The microbial richness following *A. paragallinarum* infection on the URT was evaluated at 2, 4, 7, and 9 dpi with day-0 as the baseline. Kruskal-Wallis test followed by Dunn’s test with Bonferroni correction showed that microbial richness was lower at 4 dpi compared to the baseline for both indices, Chao1 (*p* = 0.028) and Observed ASVs (*p* = 0.023), while no differences were detected at any other time point (Figure 2). The findings suggest that *A. paragallinarum* infection is associated with transient reduction in microbial richness, particularly during the acute phase infection, followed by recovery of diversity by 9 dpi. Due to the limited number of biological replicates in the longitudinal analysis, the analysis and corresponding *p*-values are descriptive rather than definitive.

**Figure 2:**
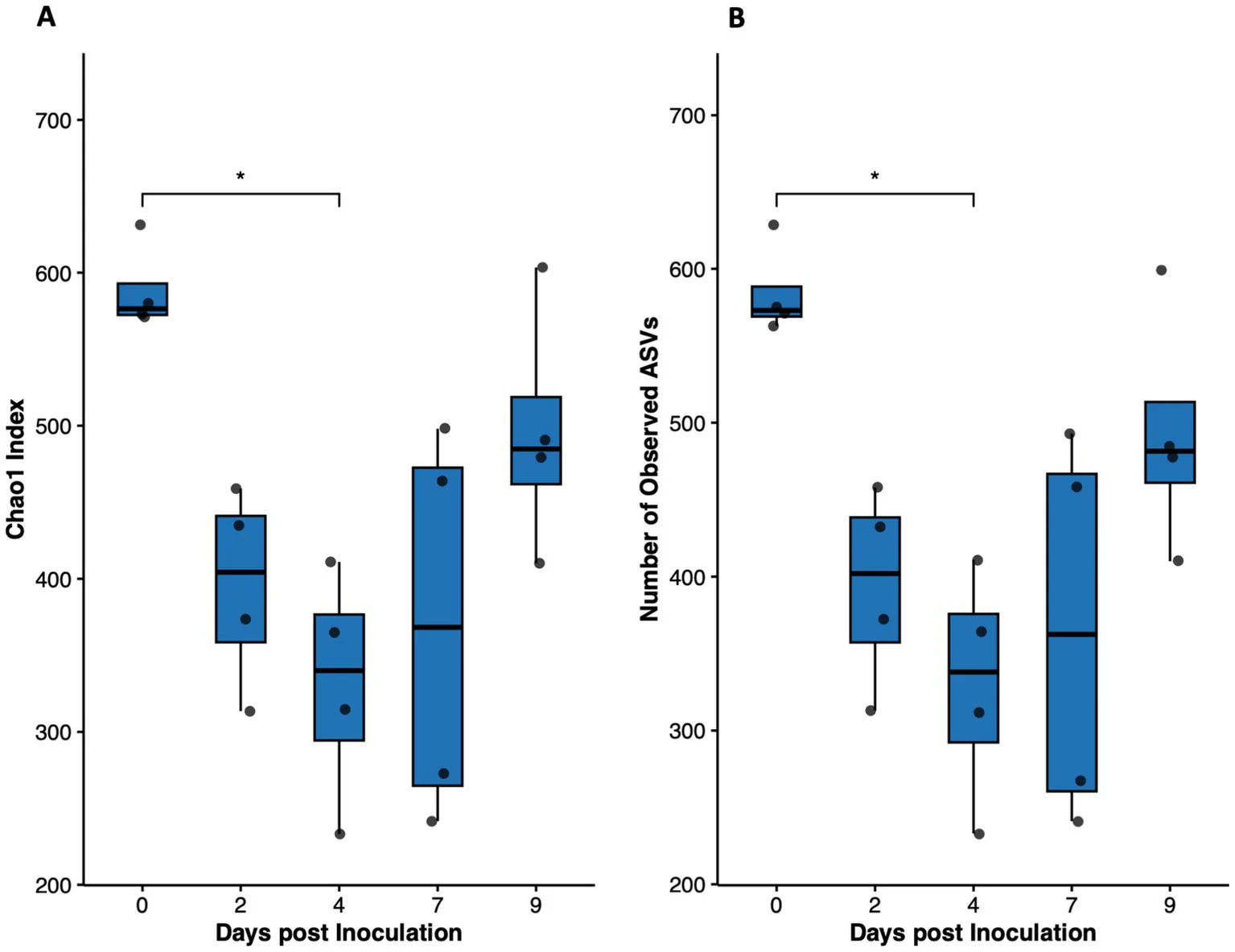
Alpha diversity metrics during *A. paragallinarum* infection. Microbial richness of the upper respiratory tract measured by Chao1 index **(A)** and Observed ASVs **(B)** at baseline (day 0) and 2, 4, 7, and 9 days post-inoculation (dpi). Boxes represent interquartile range with a central line representing median. Microbial richness was lower at 4 dpi compared to baseline for both metrics (Chao1: *p* = 0.028; Observed ASVs: *p* = 0.023; Kruskal-Wallis followed by Dunn’s test with Bonferroni correction). No differences were detected at other timepoints. Given the limited number of independent replicates, the analysis and the corresponding *p*-values should be interpreted as exploratory.

### 3.5 Microbial community dynamics

At the phylum level, the URT microbiome was dominated by Firmicutes, followed by Proteobacteria, Actinobacteria, Bacteroidetes, and other minor phyla. Following *A. paragallinarum* infection, a trend toward decreased Firmicutes and increased Proteobacteria was observed (Figure 3A). At the genus level, an uncharacterized bacterium, CHKCI001 of *Lachnospiraceae* family, was highly abundant, followed by the challenge bacterium *Avibacterium, Faecalibacterium, and Staphylococcus* in the chicken URT (Figure 3B). LEfSe analysis identified differential abundance of *Avibacterium, Lactobacillus* spp. *HT002*, and *Limosilactobacillus* in the infected group, while the noninfected group was characterized by differential abundance of *Erysipelatoclostridium*, *Butyricicoccus*, and *Lachnoclostridium* (Figure 4A). Strain-specific comparisons based on limited samples further revealed distinct taxa putatively associated with each treatment group, including *Avibacterium* in AP10, *Lactococcus* in AP36, *Salmonella* in AP37, *Staphylococcus* in AP51, and *Delftia* in control birds (Figure 4B). These differentially abundant taxa, however, represent exploratory candidates and not validated biomarkers for individual *A. paragallinarum* strains, given the limited sample size and independent biological replicates.

**Figure 3:**
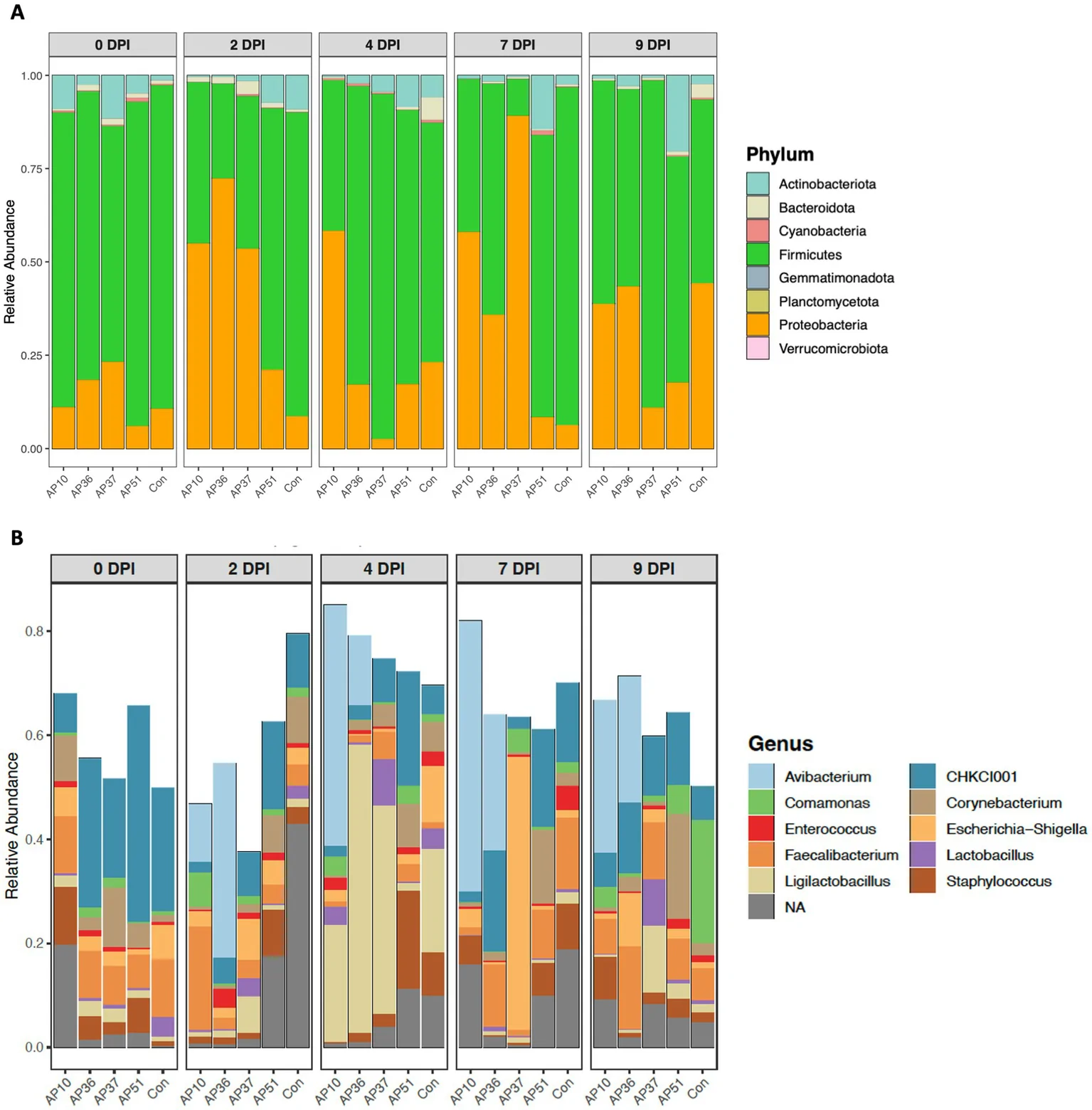
Taxonomic composition of the upper respiratory tract microbiome. **(A)** Stacked bar charts depicting the relative abundance of bacterial phyla in each treatment group (AP10, AP36, AP37, AP51, and Con) at 0, 2, 4, 7, and 9 days post-infection (DPI). **(B)** Stacked bar charts depicting the relative abundance of bacterial genera in the upper respiratory tract of chickens, showing the top 10 most abundant bacterial genera.

**Figure 4:**
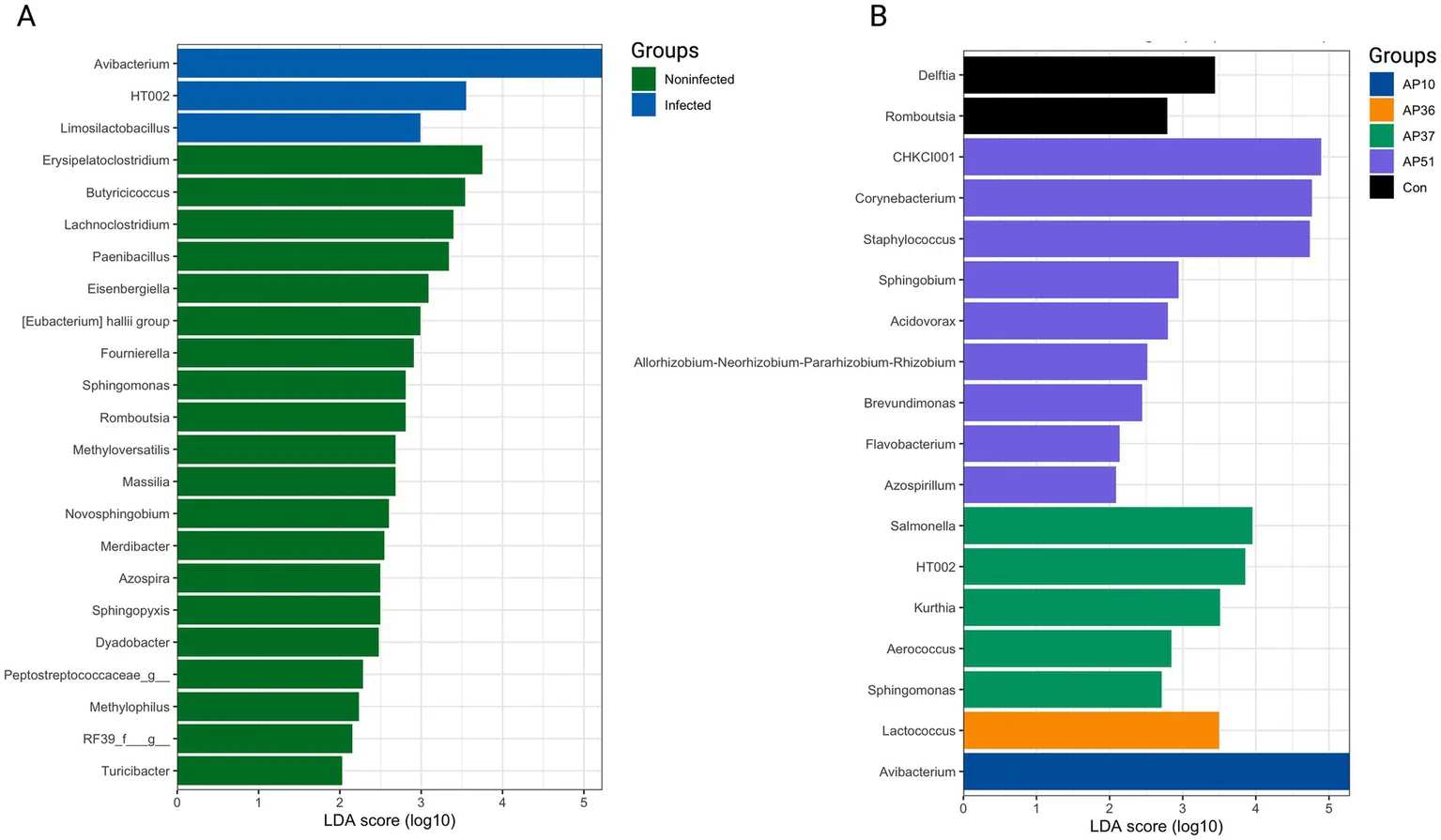
Differentially abundant taxa identified by LEfSe analysis. **(A)** Linear discriminant analysis effect size (LEfSe) results comparing the noninfected and infected chickens. Histogram of LDA scores showing taxa differentially abundant in infected groups (blue) and noninfected groups (green). A Kruskal-Wallis significance threshold of 0.05 and an LDA score cutoff (log_10_ = 2) were applied. **(B)** LEfSe analysis comparing the upper respiratory tract microbiota among different treatment groups. Histogram of LDA scores showing taxa differentially abundant in AP10 (blue), AP36 (orange), AP37 (green), AP51 (violet), and control (black) groups. The same Kruskal-Wallis threshold (0.05) and LDA score cutoff (log_10_ = 2) were applied. The differentially abundant taxa identified are exploratory candidates and not the validated strain-specific biomarkers, given the limited sample size and independent biological replicates within each strain group.

## 4 Discussion

Infection of the upper respiratory tract of the chickens caused by *A. paragallinarum* results in large economic losses in affected poultry flocks worldwide. Bacterial and viral infection of the chicken URT is known to alter the microbiota there, resulting in further negative health consequences in the chickens (17). However, the impact of *A. paragallinarum* infection on the URT microbiota is yet to be explored despite the global rise in prevalence and severity of the disease in poultry flocks. Thus, our study aimed to elucidate the impact of *A. paragallinarum* infection on the URT microbiome of SPF chickens and if the different strains influenced the microbiome distinctly. In the URT of SPF chickens, the *A. paragallinarum* infection was associated with measurable changes in microbial richness and community structure. After a transient decrease in microbial richness, it increased to the baseline after the acute phase of infection indicating a transient reduction in microbial richness.

For the challenge trial, we chose four distinct field strains of *A. paragallinarum* isolated from different chicken flocks, including two apparently non-pathogenic strains isolated from layers flocks, to assess strain-specific differences in their effects on the microbiome. The birds did not exhibit overt clinical signs of infectious coryza in any experimental group and received a clinical score of 0, including those challenged with the field strains AP10 and AP51, known to have caused clinical signs in commercial poultry flocks. The pathogenicity of *A. paragallinarum*, particularly under experimental conditions, is influenced by the route of inoculation, with intranasal instillation generally resulting in inconsistent pathogenicity compared to intra-sinus injection (32). In addition, IC pathogenicity is influenced by various factors including the age and breed of the birds, the presence of co-infecting pathogens, as well as management and environmental factors (21, 33, 34).

Our analysis indicated a decrease in microbial richness during *A. paragallinarum* infection, as evident by lower alpha diversity in the infected group compared to the noninfected group. Similar studies conducted to assess the impact of infection on the URT of chickens have also indicated the loss of microbial richness following infection with *Mycoplasma gallisepticum* (19) and avian influenza (17). Beta-diversity analyses also suggested differences in community composition between infected and noninfected groups. Differential abundance analysis indicated that the infected group has higher relative abundance of *Avibacterium*, *Lactobacillus* and *Limosilactobacillus* at the genus level. The increased relative abundance of lactic acid-producing bacteria, including *HT002* and *Limosilactobacillus*, is not easily explained with the current data but it is however, consistent with observations reported in chickens infected with low-pathogenic avian influenza (17), another respiratory disease. The increased abundance of potentially beneficial taxa such as *Lactobacillus* and *Limosilactobacillus* (35, 36) in infected groups may reflect an ecological response to acute infection, however, further studies are needed to determine the interaction of *Avibacterium* with lactobacilli in the chicken URT. Moreover, the reduced relative abundance of bacterial genera associated with beneficial butyrate-producing genera including *Butyricicoccus* and *Lachnoclostridium* in infected groups (37–39) is suggestive of microbial dysbiosis.

Previous studies on the influence of the pathogen genotype on the microbiome changes after infection are sparse and non-existent for the URT of the chickens. Thus, we investigated whether different strains of *A. paragallinarum* exerted varying effects on the URT microbiome. Alpha diversity metrics did not differ among the strain groups. While global PERMANOVA indicated an overall effect of strain in beta diversity, pairwise comparisons between individual strain groups did not reach statistical significance following *post hoc* correction, likely due to limited sample size per group. Furthermore, no distinct pattern of microbiome alteration was observed for the atypical strains compared to the clinical field strains, despite their phenotypic differences. However, the longitudinal analysis throughout the infection period indicated reduced microbial richness at 4 dpi, with a subsequent return to baseline levels by day 9. This is consistent with the previous finding about the self-limited nature of *A. paragallinarum* infection in SPF chickens, which resolves around 7 days, with bacterial load peaking at day 5 post-infection (21). Overall, the findings from the experiment indicate that *A. paragallinarum* can modulate the microbiome during infection, even in the absence of overt clinical disease.

This study has several limitations. The absence of marked clinical signs limited our ability to relate microbiome changes to disease severity; future studies may use intra-sinus injection models for the induction of more severe clinical signs. In addition, sample pooling by treatment group at each timepoint, reduced the number of independent biological replicates and statistical power; consequently, the results and associated *p*-values were interpreted as exploratory rather than confirmatory. Future studies may incorporate larger sample sizes and random sampling to visualize a finer and clearer picture of URT microbiome during *A. paragallinarum* infection. In addition, studies may incorporate serological testing alongside PCR, applied in this study to determine or validate infection status, given the widespread prevalence of non-pathogenic *A. paragallinarum* strains, which can complicate nucleic acid only based detection.

An additional limitation is the inherent resolution constraint of 16S rRNA V3-V4 amplicon sequencing, which precludes reliable classification at lower taxonomic levels. This was exemplified by the detection of a *Salmonella* ASV in the AP37 treatment group, which upon further investigation was attributed to sequence similarity among *Enterobacteriaceae* members, with the ASV attributed to *Enterobacter cloacae,* by manual blast. Closely related *Enterobacteriaceae* often share highly similar 16S rRNA sequences, such that short V3-V4 segments cannot reliably discriminate *Salmonella* from *Enterobacter* or *Citrobacter*, leading to false positive calls (40). This underscores the need for higher-resolution approaches, such as whole-genome shotgun metagenomics or full-length 16S rRNA sequencing, for accurate species-level classification of closely related taxa.

Nevertheless, the findings of this study provide valuable insights into the microbiome dynamics of the chicken upper respiratory tract under both healthy and infected states and highlight the potential impacts of *A. paragallinarum* infection on host health beyond primary pathogenicity. These insights may inform the development of targeted strategies for mitigating infectious coryza and improving overall poultry health.

## Data Availability

The datasets presented in this study can be found in online repositories. The names of the repository/repositories and accession number(s) can be found here: https://www.ncbi.nlm.nih.gov/, PRJNA1478833.

## Footnotes

1
https://benjjneb.github.io/dada2/tutorial.html
