# Supplementary material for: Impact of Avibacterium paragallinarum infection on the upper respiratory tract microbiome of chickens
Source: Front Vet Sci. 2026 Sep 21;13:1911133. doi: 10.3389/fvets.2026.1911133 (PMC13635889; doi:10.3389/fvets.2026.1911133)
Supplement: Supplementary file 1 [file Supplementary_file_1.docx]

**Supplementary Figures**

**Figure S1. Sequencing quality profiles, rarefaction curve, and read retention for microbial 16S v3-v4 rRNA gene amplicon data.** (A,B) Per‑base quality score profiles for forward (A) and reverse (B) raw reads before trimming. (C) Sample‑based rarefaction curves demonstrating saturation of ASV richness with increasing sequencing depth for individual samples. (D) Read counts retained at each major step of the DADA2 processing pipeline (input, filtering, dereplication of forward and reverse reads, merging, removal of chimeras, and final non‑chimeric reads), illustrating overall read retention throughout sequence quality control and denoising.

**Figure S2. Alpha and beta diversity of upper respiratory tract microbiota across the experimental groups challenged with different *A. paragallinarum* strains; AP10 (blue), AP36 (orange), AP37 (green), AP51 (violet), and Control (black).** (A-C) Boxplots showing alpha diversity across experimental groups based on observed ASVs (A), Chao1 richness (B), and Shannon diversity index (C); no significant differences were detected among infection groups (Kruskal-Wallis test, p > 0.05, each). (D-E) Principal coordinates analysis (PCoA) based on weighted (D) and unweighted (E) UniFrac distances illustrating beta-diversity patterns among experimental groups, with 95% confidence ellipses for each group. Global PERMANOVA showed a significant overall effect of strain group on community composition (p < 0.05, each); pairwise comparisons, however, were not significant after Bonferroni correction (p > 0.05, each). Statistical analysis and the associated p-values are exploratory rather than confirmatory.
